# The association between antibiotics and community-associated *Staphylococcus aureus* colonization in the United States population: Analysis of the National Health and Nutrition Examination Survey (NHANES)

**DOI:** 10.1097/MD.0000000000031702

**Published:** 2022-11-11

**Authors:** Baixing Chen, Shaoshuo Li, Shi Lin, Mingling Huang, Hang Dong

**Affiliations:** a Department of Development and Regeneration, KU Leuven, Leuven, Belgium; b Nanjing University of Chinese Medicine, Nanjing, Jiangsu Province, China; c Guangzhou University of Chinese Medicine, Guangzhou, Guangdong Province, China; d Department of traumatology, The First Affiliated Hospital, Guangzhou University of Chinese Medicine, Guangzhou, Guangdong Province, China.

**Keywords:** antibiotics, broad-spectrum antibiotics, colonization, methicillin-resistant *Staphylococcus aureus*, NHANES, *Staphylococcus aureus*

## Abstract

*Staphylococcus aureus* nasal colonization is a seriously opportunistic infection. However, there is a lack of research of relationship between antibiotics and *S aureus* colonization in the general population. Through a cross-sectional investigation, this study intends to establish the parameters related to *S aureus* nasal colonization, specifically the function antibiotics play in colonization. The key information from 2001 to 2004 was abstracted from National Health and Nutrition Examination Survey (NHANES), including information on general demographics, health care status, antibiotic prescription, diabetes, alcohol consumption, and tobacco smoke exposure. The participants colonized with methicillin-susceptible *S aureus* (MSSA), or methicillin-resistant *S aureus* (MRSA) were defined as the case group, and the control group was subjects without positive *S aureus* colonization. Univariate and multivariate logistic regression models were used to identify the variables associated with MSSA and MRSA colonization. The records of 18,607 individuals were included, involving 13,205 cases without *S aureus* colonization, 5195 cases with MSSA, and 207 cases with MRSA. In the multivariate logistic regression analysis, the risk of MSSA colonization was significantly reduced with fluoroquinolone use (75% risk reduction, *P* = .02), sulfonamide use (98% risk reduction, *P* < .01), tetracycline use (81% risk reduction, *P* < .01) and antibiotic combination therapy (risk reduction 76%, *P* < .01). Female, race and total household size were strongly associated with MSSA carriage. On the other hand, regarding MRSA colonization, fluoroquinolone use, long-term care, and former smoker were positively associated with MRSA colonization, while high income was negatively associated with MRSA colonization. More proper use of broad-spectrum antibiotics contributes to reducing MSSA colonization. Former smokers should also practice better personal hygiene to limit the possibility of MRSA colonization.

## 1. Introduction

*Staphylococcus aureus* colonization is significantly associated with many infectious illnesses, including endocarditis, periarticular infections, and postoperative infections.^[1,2]^ The proportions of colonization caused by methicillin-resistant *S aureus* (MRSA) strains, on the other hand, has been growing during the last few decades. Every year, it kills around 19,000 inpatients in the United States.^[3,4]^ Furthermore, *S aureus* nasal colonization highly linked to recurrent infections in individuals. Although there have been numerous research on *S aureus* colonization, the majority of them have focused on patients with a specific condition (e.g., dialysis patients and HIV patients)^[5,6]^ or on specific special occupational groups.^[7,8]^ Just a few studies have investigated the characteristics of *S aureus* colonization in community populations. However, the sample sizes of in these studies are too small or the respondents are not representative, preventing them from answering the research questions accurately.^[9–11]^

The community prevalence of *S aureus* is increasing due in large part to the colonization of *S aureus*.^[12,13]^ Antibiotics usages is a known factor that influences *S aureus* colonization. However, whether the antibiotic is a danger or a protective factor in *S aureus* colonization yields contradictory data.^[9,14–16]^ Most investigations have only been conducted in a few places and with unrepresentative people.^[11,17]^ Furthermore, most studies on the problem have been limited to using antibiotics as a factor without classifying them based on their mode of action in bacteria for analysis. A similar scenario exists in MRSA colonization studies. Given the serious consequences of *S aureus* and MRSA infection, earlier identification of high risk carriages is thought to be critical for reducing the overall prevalence of *S aureus* infection.

The National Health and Nutrition Examination Survey (NHANES) collects data on the health and nutrition of a large, nationally representative sample of the United States population. Discovering the epidemiology and risk factors of *S aureus* and MRSA carriage is crucial, but the influence of antibiotics on *S aureus* colonization in people of all ages in the United States has yet to be fully recognized. Through a cross-sectional investigation, this study intends to establish the parameters related to *S aureus* nasal colonization, specifically the function antibiotics play in colonization.

## 2. Method

### 2.1. Data source

The NHANES was used to assess the health and nutritional status of the populations in the United States. Each year, NHANES randomly selects permanent residents with American citizenship from various regions of the United States to gather various forms of demographic data, including questionnaire surveys, medical examinations and laboratory tests. The key information from 2001 to 2004 was abstracted in order to investigate the risk factors for *S aureus* and methicillin-resistant *S aureus* (MRSA) colonization in community populations. Participants were excluded based on the following criteria: missing or invalid pathogen data; and participants who did not know the name of the medication or refused to report the name of the medication. The NCHS Research Ethics Review Board approved the surveys in the NHANES protocol for 2011 to 2017. All methods were performed according to the relevant guidelines and regulations and all participants or their proxy provided written informed consent.

### 2.2. Ascertainment of medication use data

The questionnaire determined prescription medication information was determined by the questionnaire (In the past 30 days have you used medication for which a prescription is needed?), and the information was collected from prescription medication containers or participants who verbally reported this information if the container was not available. Antibiotics were classified using the Multum Lexicon Plus drug database.^[18]^ We examined all medications in the first-level category, except topical antibiotics. We then generated variables for twelve different antibiotic categories using the Multum Lexicon Drug Database’s second-level drug categories, including: cephalosporins, macrolides, penicillins, fluoroquinolones, sulfonamides, tetracyclines, aminoglycosides, lincomycin derivatives, glycopeptide antibiotics, antibiotics unspecified and antibiotic combination therapy (i.e., a combination of 2 or more antibiotics as mentioned above). Participants who had non-antibiotic treatment alone were chosen as non-antibiotic medications to minimize their low loss. Furthermore, NHANES did not specify the mode of administration or any indications for the application of medications.

### 2.3. Outcome measurement of SA and MRSA

Nasal swabs from eligible individuals aged 1 year and older were tested for *S aureus* colonization during the survey’s physical examination portion. Standard culture-based techniques were then used to assess the presence of *S aureus* in nasal swabs. Further information on the *S aureus* screen test can be found in the description of laboratory methodology on the NHANES website.^[18]^ Disk diffusion was employed to test identified *S aureus* isolates for methicillin resistance. Participants who tested negative for *S aureus* were also included in the control group. Those who tested positive for *S aureus* but not MRSA were labeled as having methicillin-susceptible *S aureus* (MSSA). Those who tested positive for MRSA were labeled as such. In addition, no information on clinical infection status were gathered.

### 2.4. Covariates measurement

The demographic data comprised gender, age, education level, family income and the total number of individuals in the household as covariates for the multivariate analysis. Non-Hispanic white, Mexican, other Hispanic, non-Hispanic black, and the others were the race categories. Education was categorized as less than high school, high school, and more than high school, and with individuals under the age of 12 classified as less than high school. Income was coded dichotomously (earning <$45,000 a year). The total number of individuals in the household was represented as a continuous variable. In addition, various *S aureus* related health indicators were gathered, such as insurance status, time spent in long-term care facilities in the pervious 12 months, current health status and respiratory health. The questionnaire identified the current health state (“Did you have flu, pneumonia, or ear infections that started during those 30 days?”) and was dichotomized. Respiratory health was determined from the questionnaire (“Do you usually cough on most days for 3 consecutive months or more during the year?” and “Do you bring up phlegm on most days for 3 consecutive months or more during the year?”). Furthermore, various risk factors were included as covariates in the analysis, including the use of insulin, which was modeled as a dichotomous variable. Diabetes mellitus^[19]^ was also modeled as a categorical variable involving verified diabetes, no diabetes, and borderline diabetes. Participants were divided into 3 groups: never-smokers (those who have smoked <100cig/lifetime), former smokers (having smoked > 100cig/lifetime but do not currently smoke), and current smokers. All of the information was derived from self-reported questionnaires responses.

### 2.5. Survey weights

In order to account for the complex survey design of NHANES, the sampling weights are analyzed in accordance with the guidelines provided by the National Center for Health Statistics. We employed Mobile Examination Center survey weight to alter our estimates for each model in order to reduce the population’s ultimate unbiased.

### 2.6. Statistical analysis

The category variables were described using a number (percentage). Continuous variables were classified as normally distributed or non-normally distributed, using mean ± standard deviation and median (interquartile range) to define normally distributed variables and non-normally distributed variables, respectively. The chi-square test for categorical variables, the *t* test for normally distributed variables and the Mann–Whitney *U* test for non-normally distributed variables were used to make comparisons between participants without *S aureus* colonization and those with MSSA or MRSA.

The relationship between antibiotics and MRSA/MSSA colonization was analyzed by using univariate and multivariate logistic regression models. After calculating all variables using univariate logistic regression, variables with *P* values < .20 were included in the multivariate logistic regression analysis, which was then performed using backward-stepwise approaches to determine independent factors and manually remove them one by one. All factors were estimated as odds ratios (ORs) and 95% confidence intervals (CIs), and all significant associations were examined. The *P* value of <.05 indicated that the difference was statistically significant. The survey package and the logistics regression models were used to create the survey design object.^[19,20]^ All analyses were performed using R (version 4.1.1).

## 3. Result

### 3.1. Subject characteristics

From 2001 to 2004, the NHANES database included information on 21,161 participants. Participants who did not test positive for *S aureus* or did not have prescription medication information were excluded from the research. Finally, the records of 18,607 individuals were included, involving 13,205 cases without *S aureus* colonization, 5,19 5cases with MSSA, and 207 cases with MRSA. Table 1 shows descriptive statistics indicating that the prevalence of MSSA and MRSA was 27.9% and 1.1%, respectively.

**Table 1:** Weighted characteristics and distribution of anthropometric measures in study sample.

Variable	Control	MSSA^†^	MRSA^‡^	MSSA vs control	MRSA vs control
				*P* value	*P* value
	n = 13205	n = 5195	n = 207		
**Gender**				<.001*	.132
Male	6201 (47.0)	2793 (53.8)	93 (44.9)		
Female	7004 (53.0)	2402 (46.2)	114 (55.1)		
**Age**				<.001*	.439
0–20	6196 (46.9)	2828 (54.4)	79 (38.2)		
21–40	2449 (18.5)	938 (18.1)	29 (14.0)		
41–60	2049 (15.5)	739 (14.2)	22 (10.6)		
>60	2511 (19.0)	690 (13.3)	77 (37.2)		
**Race**				.001*	.316
Mexican American	3334 (25.2)	1287 (24.8)	33 (15.9)		
Other Hispanic	471 (3.6)	265 (5.1)	2 (1.0)		
White^§^	5291 (40.1)	2253 (43.4)	102 (49.3)		
Black	3549 (26.9)	1195 (23.0)	63 (30.4)		
Other race^¶^	560 (4.2)	195 (3.8)	7 (3.4)		
**Education**				<.001*	.069
<High school diploma	6057 (45.9)	2753 (53.0)	92 (44.4)		
High school diploma	1967 (14.9)	694 (13.4)	30 (14.5)		
>High school diploma	3315 (25.1)	1239 (23.8)	54 (26.1)		
**Number of household** ^#^				<.001*	.257
1–3	6094 (46.1)	2033 (39.1)	121 (58.5)		
≥4	7111 (53.9)	3162 (60.9)	86 (41.5)		
**Household income** (USD)				**.002**	.426
<45,000	7321 (55.4)	2734 (52.6)	152 (73.4)		
≥45,000	4886 (37.0)	2095 (40.3)	42 (20.3)		
**Antibiotics class**				.175	.548
No medicine	7942 (60.1)	3290 (63.3)	86 (41.5)		
Cephalosporins	100 (0.8)	37 (0.7)	0 (0.0)		
Macrolides	87 (0.7)	15 (0.3)	0 (0.0)		
Penicillins	309 (2.3)	125 (2.4)	5 (2.4)		
Fluoroquinolones	36 (0.3)	5 (0.1)	3 (1.4)		
Sulfonamides	33 (0.2)	1 (0.0)	0 (0.0)		
Tetracyclines	94 (0.7)	9 (0.2)	0 (0.0)		
Aminoglycosides	3 (0.0)	2 (0.0)	1 (0.5)		
Lincomycin derivatives	18 (0.1)	2 (0.0)	0 (0.0)		
Antibiotic combination therapy	82 (0.6)	11 (0.2)	2 (1.0)		
Antibiotics unspecified	49 (0.4)	15 (0.3)	0 (0.0)		
Non-antibiotics drugs	4452 (33.7)	1683 (32.4)	110 (53.1)		
**Coughing** ^##^				.125	.090
No	9389 (71.1)	3575 (68.8)	139 (67.1)		
Yes	726 (5.5)	217 (4.2)	18 (8.7)		
**Phlegm** ^###^				.115	.161
No	9379 (71.0)	3554 (68.4)	131 (63.3)		
Yes	737 (5.6)	237 (4.6)	26 (12.6)		
**Current health status** ^####^				.016*	.073
No	11592 (87.8)	4625 (89.0)	180 (87.0)		
Yes	794 (6.0)	257 (4.9)	11 (5.3)		
**Insurance**				.041*	.129
No	9899 (75.0)	3904 (75.1)	164 (79.2)		
Yes	684 (5.2)	288 (5.5)	13 (6.3)		
**Long-term care**				.052	.169
No	13114 (99.3)	5174 (99.6)	195 (94.2)		
Yes	90 (0.7)	21 (0.4)	12 (5.8)		
**Diabetes mellitus**				.013*	.200
No	12394 (93.9)	4903 (94.4)	179 (86.5)		
Borderline	110 (0.8)	34 (0.7)	4 (1.9)		
Yes	695 (5.3)	257 (4.9)	24 (11.6)		
**Insulin**				.008*	.053
No	13028 (98.7)	5128 (98.7)	202 (97.6)		
Yes	177 (1.3)	67 (1.3)	5 (2.4)		
**Alcohol consumption** (drink/yr)				.151	.078
<12	2078 (15.7)	674 (13.0)	42 (20.3)		
≥12	4436 (33.6)	1510 (29.1)	74 (35.7)		
**Smoking status**				.147	.389
Never-smokers	5582 (42.3)	2289 (44.1)	64 (30.9)		
Former smokers	1922 (14.6)	655 (12.6)	58 (28.0)		
Current smokers	2373 (18.0)	754 (14.5)	32 (15.5)		

† MSSA - Methicillin-susceptible *Staphylococcus aureus*.

‡ MRSA - Methicillin-resistant *Staphylococcus aureus*.

§ Non-Hispanic White.

Non-Hispanic Black.

¶ Other Race - Including Multi-Racial.

# The total number of people in the household.

## Coughing over 3 months period.

### Bring up phlegm over 3 months period.

#### having flu, pneumonia, or ear infections that started during those 30 days.

*P < .05.

The characteristics of the study sample as determined by the weighted analysis approach are shown in Table 1. The participants with MSSA had a lower mean age (27.51 ± 22.34 years) than the controls (31.51 ± 24.8 years). For MSS carriage, the total number of individuals in the household was higher than the controls (4.01 ± 1.71 vs 3.79 ± 1.74). In comparison to the control group, the MSSA group had a higher proportion of males (53.8% vs 47.0%), whites (43.4% vs 40.1%), and an income greater than 45,000 (40.3% vs 37.0%). MSSA participants had considerably lower rates of influenza, pneumonia, and ear infections than controls. It also revealed that people with MSSA had a much higher proportion of insurance than those in the control group.

In contrast, the participants with MRSA had a greater mean age (40.13 ± 29.14 years) than the mean age of the controls (31.51 ± 24.8 years). Males (44.9% vs 47.0%) and Mexican Americans (15.9% vs 24.8%) had a lower proportion of MRSA carriages than controls. Participants with MRSA had a greater rate of coughing (8.7% vs 5.5%), bringing up phlegm (12.6% vs 5.6%), long term care (5.8% vs 0.7%), diabetes mellitus (11.6% vs 5.3%), and being a former smoker (28.0% vs 14.6%) than those from the control group. Univariate analyses of MSSA/ MRSA cases versus controls

There were significant differences (*P* < .20) between MSSA cases and controls in age, gender, race, education, total number of people in the household, household income, macrolides use, fluoroquinolones use, sulfonamide use, tetracycline use, lincomycin derivatives use, antibiotic combination therapy, coughing, long term care, smoking status in univariate analyses (Table 2). MRSA carriage was associated with significant variations in age, gender, race, total number of individuals in the household, fluoroquinolones use, bringing up phlegm, long term care, diabetes, and former smokers compared to the control group. Variables with substantial statistics were used to construct multivariable regression models. Multivariate analyses of MSSA cases versus controls

**Table 2:** Univariate analyses of MSSA/MRSA cases versus controls by weighted.

Variable	Control vs MSSA^†^		Control vs MRSA^‡^	
	*OR* (95% *CI*)	*P* value	*OR* (95% *CI*)	*P* value
**Gender**				
Female	0.72 (0.65–0.79)	<.01*	1.31 (0.94–1.82)	.13*
**Age**				
0–20	Reference		Reference	
21–40	0.76 (0.69–0.83)	<.01*	0.90 (0.59–1.38)	.65
41–60	0.73 (0.65–0.84)	<.01*	0.84 (0.40–1.77)	.66
>60	0.55 (0.49–0.61)	<.01*	2.48 (1.43–4.29)	<.01*
**Race**	1.01 (0.89–1.15)			
Mexican American	Reference		Reference	
Other Hispanic	1.41 (1.18–1.67)	<.01*	0.27 (0.05–1.54)	.15*
White^§^	1.12 (0.99–1.26)	.08*	1.98 (0.90–4.39)	.10*
Black	0.78 (0.71–0.87)	<.01*	1.86 (0.89–3.90)	.11*
Other Race^¶^	0.89 (0.69–1.14)	.37	1.27 (0.44–3.65)	.66
**Education**				
<High School Diploma	Reference		Reference	
High School Diploma	0.74 (0.66–0.83)	<.01*	1.02 (0.73–1.42)	.91
>High School Diploma	0.83 (0.76–0.92)	<.01*	0.87 (0.57–1.33)	.54
**Number of Household** ^#^				
1–3	Reference		Reference	
≥4	1.33 (1.20–1.47	<.01*	0.59 (0.34–1.03)	.07*
**Household income** (USD)				
<45,000	Reference		Reference	
≥45,000	1.17 (1.06–1.29)	<.01*	0.42 (0.26–0.66)	<.01*
**Prescription medicine**				
No medicine	Reference		Reference	
Cephalosporins	0.88 (0.58–1.32)	.53	0	0
Macrolides	0.53 (0.30–0.91)	.03*	0	0
Penicillins	1.09 (0.80–1.48)	.59	1.61 (0.48–5.35)	.45
Fluoroquinolones	0.07 (0.02–0.30)	<.01*	7.14 (1.74–29.27)	.01*
Sulfonamides	0.08 (0.03–0.21)	<.01*	0	0
Tetracyclines	0.20 (0.08–0.46)	<.01*	0	0
Aminoglycosides	0.89 (0.06–14.29)	.93	5.81 (0.50–67.09)	.18
Lincomycin derivatives	0.16 (0.03–0.99)	.06*	0	0
Antibiotic combination therapy	0.15 (0.06–0.41)	<.01*	1.76 (0.23–13.52)	.59
Antibiotics unspecified	0.52 (0.24–1.14)	.12	0	0
Non-antibiotics drugs	0.93 (0.85–1.01)	.09*	2.55 (1.78–3.67)	<.01*
**Coughing** ^##^				
Yes	0.78 (0.62–0.99)	.05*	1.34 (0.73–2.45)	.35
**Phlegm** ^###^				
Yes	0.90 (0.72–1.12)	.35	1.72 (0.97–3.04)	.07*
**Current health status** ^####^				
Yes	0.96 (0.78–1.17)	.68	1.34 (0.49–3.66)	.57
**Insurance**				
Yes	1.05 (0.89–1.25)	.58	1.66 (0.78–3.54)	.20
**Long-term care**				
Yes	0.48 (0.25–0.90)	.03*	4.33 (2.02–9.29)	<.01*
**Diabetes mellitus**				
No	Reference		Reference	
Borderline	1.12 (0.60–2.06)	.73	2.62 (0.91–7.53)	.09*
Yes	0.97 (0.75–1.24)	.80	1.94 (1.14–3.31)	.02*
**Insulin**				
Yes	1.08 (0.69–1.68)	.75	1.53 (0.53–4.45)	.44
**Alcohol consumption** (drink/yr)				
<12	Reference		Reference	
≥12	1.06 (0.92–1.23)	.42	0.86 (0.52–1.41)	.55
**Smoking status**				
Never-smokers	Reference		Reference	
Former smokers	0.90 (0.79–1.03)	.12*	2.58 (1.53–4.36)	<.01*
Current smokers	0.78 (0.70–0.86)	<.01*	1.13 (0.62–2.06)	.70

CI = confidence interval, OR = odds ratio.

† MSSA - Methicillin-susceptible *Staphylococcus aureus*.

‡ MRSA - Methicillin-resistant *Staphylococcus aureus*.

§ Non-Hispanic White.

Non-Hispanic Black.

¶ Other Race - Including Multi-Racial.

# The total number of people in the household.

## Coughing over 3 months period.

### Bring up phlegm over 3 months period.

#### having flu, pneumonia, or ear infections that started during those 30 days.

*P*** ***< .20.

In the multivariate logistic analysis (Table 3), MSSA colonization was inversely correlated with advanced age (*P* < .01). Female (odds ratio [OR] = 0.69, 95% CI [0.62–0.77], *P* < .01), non-Hispanic Black (OR = 0.84, 95% CI [0.77–0.93], *P* < .01) and current smokers (OR = 0.73, 95% CI [0.65–0.81], *P* < .01) were inversely associated with having MSSA colonization. Furthermore, the risk of MSSA colonization was significantly reduced with fluoroquinolone use (risk reduction 75%, OR = 0.25, 95% CI [0.09–0.72], *P* = .02), sulfonamide use (risk reduction 98%, OR = 0.02, 95% CI [0.01–0.13], *P* < .01), tetracycline use (risk reduction 81%, OR = 0.19, 95% CI [0.08–0.46], *P* < .01), and antibiotic combination therapy (risk reduction 76%, OR = 0.24, 95% CI [0.12–0.47], *P* < .01). Factors associated with a higher risk of MSSA carriage were Other Hispanic (OR = 1.51, 95% CI [1.27–1.80], *P* < .01), Non-Hispanic White (OR = 1.29, 95% CI [1.14–1.46], *P* < .01), and the total number of individuals in the household (OR = 1.19, 95% CI [1.06–1.33], *P* < .01). The probability of MSSA carriage associated with education, income, coughing, long term care, and former smokers seen in univariable analysis was not found in multivariable analysis.

**Table 3:** Multivariate analyses of MSSA/MRSA cases versus controls.

Variable	Control vs MSSA				Control vs MRSA			
	Unadjusted model^†^		Final adjusted model^‡^		Unadjusted model^†^		Final adjusted model^§^	
	OR (95% CI)	*P* value	OR (95% CI)	*P* value	OR (95% CI)	*P* value	OR (95% CI)	*P* value
**Gender**								
Female	0.69 (0.62–0.77)	<.01*	0.69 (0.62–0.77)	<.01*	1.31 (0.94–1.84)	.13		
**Age**								
0–20	Reference		Reference		Reference			
21–40	0.78 (0.66–0.93)	.01*	0.82 (0.72–0.93)	.01*	0.95 (0.48–1.87)	.89		
41–60	0.71 (0.62–0.83)	<.01*	0.74 (0.65–0.85)	<.01*	0.71 (0.32–1.55)	.40		
>60	0.54 (0.45–0.64)	<.01*	0.55 (0.47–0.63)	<.01*	1.35 (0.64–2.86)	.44		
**Race** (%)								
Mexican American	Reference		Reference		Reference			
Other Hispanic	1.50 (1.25–1.80)	<.01*	1.51 (1.27–1.80)	<.01*	0.25 (0.04–1.40)	.12		
White	1.27 (1.11–1.45)	<.01*	1.29 (1.14–1.46)	<.01*	1.91 (0.77–4.71)	.17		
Black^¶^	0.82 (0.74–0.91)	<.01*	0.84 (0.77–0.93)	<.01*	1.79 (0.82–3.89)	.15		
Other Race^#^	0.92 (0.71–1.19)	.53	0.95 (0.73–1.22)	.67	1.37 (0.45–4.18)	.59		
**Education** (%)								
<High School Diploma	Reference							
High School Diploma	1.00 (0.86–1.17)	.99						
>High School Diploma	1.09 (0.92–1.28)	.32						
**Number of Household** ^#^								
1–3	Reference		Reference		Reference			
≥4	1.18 (1.05–1.32)	**.01**	1.19 (1.06–1.33)	**.01**	0.90 (0.44–1.85)	.78		
**Household income** (USD)								
<45,000	Reference				Reference			
≥45,000	1.01 (0.91–1.12)	.89			0.50 (0.32–0.78)	<.01*	0.42 (0.27–0.67)	<.01*
**Prescription medicine** (%)								
No medicine	Reference				Reference		Reference	
Cephalosporins	0.90 (0.58–1.39)	.63			0	-		
Macrolides	0.56 (0.31–0.99)	.05			0	-		
Penicillins	1.02 (0.73–1.41)	.92			1.50 (0.43–5.21)	.53		
Fluoroquinolones	0.26 (0.09–0.76)	.02*	0.25 (0.09–0.72)	.02*	5.28 (1.22–22.82)	**.03**	5.90 (1.37–25.33)	**.02**
Sulfonamides	0.02 (0.01–0.13)	<.01*	0.02 (0.01–0.13)	<.01*	0	-		
Tetracyclines	0.19 (0.08–0.46)	<.01*	0.19 (0.08–0.46)	<.01*	0	-		
Aminoglycosides	0.50 (0.03–9.77)	.65			2.65 (0.17–42.17)	.50		
Lincomycin derivatives	0.19 (0.03–1.31)	.10			0	-		
Antibiotic combination therapy	0.24 (0.12–0.48)	<.01*	0.24 (0.12–0.47)	<.01*	1.68 (0.25–11.11)	.59		
Antibiotics unspecified	0.81 (0.38–1.70)	.58			0	-		
Non-antibiotics drugs	1.16 (1.04–1.29)	.01*	1.16 (1.03–1.29)	.02*	2.28 (1.49–3.49)	<.01*	2.51 (1.75–3.60)	<.01*
**Coughing** ^#^								
Yes	0.85 (0.67–1.07)	.17			1.27 (0.66–2.44)	.49		
**Long-term care** (%)								
Yes	0.61 (0.33–1.12)	.12			2.54 (1.21–5.33)	**.02**	2.86 (1.37–5.95)	**.01**
**Insurance**								
Yes					2.15 (0.99–4.65)	.06		
**Smoking status**								
Never-smokers	Reference				Reference			
Former smokers	0.93 (0.81–1.08)	.34			2.07 (1.18–3.66)	**.02**	2.11 (1.26–3.55)	**.01**
Current smokers	0.75 (0.68–0.84)	<.01*	0.73 (0.65–0.81)	<.01*	1.04 (0.55–1.98)	.90		
**Diabetes mellitus** (%)								
No					Reference			
Borderline					1.48 (0.45–4.85)	.52		
Yes					1.11 (0.64–1.94)	.71		

CI = confidence interval, OR = odds ratio.

† Unadjusted model, multivariable full models correspond to models including all the variables with *P* value < 0.20 in univariate analyses.

‡ Final adjusted models for gender, age, race, the total number of people in the household, prescription medicine and smoking status, which correspond to models including all the remaining variables showing significant OR estimates after manual backward-stepwise selections.

§ Final adjusted models for race, household income, fluoroquinolones, long-term care and former smokers, which correspond to models including all the remaining variables showing significant OR estimates after manual backward-stepwise selections.

Non-Hispanic White.

¶ Non-Hispanic Black.

# Other Race - Including Multi-Racial.

## The total number of people in the household.

*P < .05.

### 3.2. Multivariate analyses of MRSA cases versus controls

Long-term care (OR = 2.86, 95% CI [1.37–5.95], *P* = .01) and former smokers (OR = 2.11, 95% CI [1.26–3.55], *P* = .01) were factors associated with a higher risk of MRSA colonization. Participants using fluoroquinolones have a nearly 6-fold increased risk of MRSA colonization (OR = 5.90, 95% CI [1.37–25.33], *P* = .02). More than 45,000 USD in household income (OR = 0.42, 95% CI [0.27–0.67, *P* < .01) was negatively associated with MRSA (Table 3).

## 4. Discussion

The goal of this study is to identify the high-risk factors, for *S aureus* nasal colonization, especially the connection with antibiotics, through a cross-sectional investigation. The NHANES records of 18,607 individuals met the inclusion criteria. In this study, the prevalence of MSSA was 27.9% and that of MRSA was 1.1%. MSSA colonization was found to be significantly associated with advanced age. The results indicated that females, non-Hispanic blacks, and current smokers had a lower probability of MSSA carriage. On the other hand, the risk of MSSA colonization was significantly reduced in individuals taking fluoroquinolones, sulfonamides, tetracyclines and antibiotic combination therapy, with a 74% to 98% risk reduction. Long-term care, former smokers and fluoroquinolone usage all elevated the probability of carriage, with fluoroquinolone use raising the risk of colonization by 6-fold. Surprisingly, a household income of more than 45,000 USD was found to inversely associated with MRSA colonization. While population-based research cannot be utilized to investigate causality, some of these characteristics related to MRSA colonization can be modified on an individual or societal level.

The key discovery of this analysis was the association between broad-spectrum antibiotics and MSSA colonization. The relative relevance of antibiotics in *S aureus* colonization is contested in the literature. Nguyen et al found that antibiotic use to be an independent explanatory variable for *S aureus* carriage, with an OR (0.69) indicating a negative connection between them.^[9]^ A cross-sectional study by Chen et al^[21]^ argued against this, stating that antibiotics exposure in the previous 4 weeks had no significant effect on carriage rates. This conclusion was also agreed upon by Munckhof et al.^[15]^ To date, no plausible explanation for these contradictory observations has been proposed. This could be owing to a lack of antibiotics classification and the possibility of selection bias in these investigations.^[22]^ MSSA colonization is reduced by broad-spectrum bactericidal efficacy and selection pressure from diverse drug. Uncontrolled use of antibiotics is the main reason why some studies consider antibiotics to be a risk factor for colonization. In contrast, the antibiotics used by NHANES subjects were prescription medications, and the majority of them were monitored. Therefore, we conclude that the controlled and proper use of antibiotics is a protective factor against *S aureus* colonization. However, due to the lack of antibiotic dose information in NHANES, we were unable to investigate them in further depth.

Another interesting discovery is the relationship between overall household size and MSSA colonization. Families have been demonstrated to act as community reservoirs of *S aureus*.^[23]^ Following an infected family member, high levels of *S aureus* colonization are common among other family members, especially in large families with large populations.^[24,25]^ Our findings are consistent prior research that suggests MSSA carriage may be caused by living in more crowded conditions, which increases the chance of *S aureus* transmission. Age was also found to be a substantial predictor of MSSA. We discovered that the probability of MSSA carriage reduces with age (OR value decreases consistently), which is consistent with the finding of earlier investigations.^[9,26]^In our study, current smoking was found to be inversely related to the risk of MSSA colonization. Other investigations have observed similar findings, with the hypothesis that cigarettes have bactericidal activity and increase hypoxia-related immunoreactivity.^[27,28]^ Furthermore, the risk of MSSA carriage varies by race, with whites and other Hispanics being more at risk than blacks.^[26]^ We cannot assume that race is connected with limited access to healthcare because race cannot be used as a proxy for socioeconomic situations and no statistical differences were detected at the education and income levels.^[29]^

We found no consistency in risk variables for MRSA and MSSA colonization in community populations. However, 2 characteristics, smoking and quinolone use, were found to be associated in different directions with MRSA and MSSA colonization. Tobacco exposure, in particular, reduces the growth of colonizing bacteria, which could explain why being exposed to tobacco is a protective factor for MSSA.^[30]^ Simultaneously, tobacco smoking causes DNA damage to the induced *S aureus* in the nasal cavity, hastening the pace of mutation and eventually leading to antibiotic resistance. Former smokers may become factors for MRSA colonization due to a lack of tobacco suppression and MRSA being the primary colonizing bacteria in the nasal cavity. During this time, tobacco exposure-induced impairment of mucociliary clearance mechanisms in the sinus epithelium, as well as down-regulation of local immunological responses, both enhance MRSA colonization and subsequent reinfection.^[31]^ Additionally, the usage of fluoroquinolones was strongly associated with MRSA but negatively associated with MSSA colonization. Previous research has found that using fluoroquinolones increases the probability of MRSA colonization^[32]^ or infection^[33]^ when compared to no *S aureus* isolation. It has been proposed that due to their excellent tissue diffusion, fluoroquinolones can encourage the proliferation and selection of MRSA by removing fluoroquinolone-sensitive microbe such as MSSA.^[34]^ However, due to only 3 subjects using fluoroquinolones in the MRSA group, this has to be interpreted with caution.

Furthermore, we discovered 2 risk factors for MRSA colonization: long-term care and higher income. Subjects in long-term care facilities have previously been found to have higher MRSA colonization, a risk factor that may be a sign of disease severity and suggests the probability of bacterial transmission through caregivers. Economic pressures increased the likelihood of taking antibiotics in Sweden,^[30]^ whereas in the United Kingdom,^[35]^ higher rates of antibiotic prescribing were reported in primary care in poorer areas. Although we cannot conclude that income increases or decrease the odds of antibiotic base on this study, income was substantially connected with MRSA and high income was a protective factor for MRSA. This could be due to the relationship between income and the level of care.

A limitation of this study is that the demographic data from NHANES was obtained 15 years ago. Despite the fact that it was the only national data available, it is possible the epidemiological changes may not reflect *S aureus*. Second, different ways of evaluating of *S aureus* colonization, such as study design, control group definition and population,^[33]^ may have an impact on the evaluation of *S aureus* colonization. For example, we may overstate the importance and risk of antibiotics in the carriage of MRSA if we utilize MSSA as a control group. Third, non-antibiotic medicines have the potential to be relevant to MRSA carriage. Unfortunately, the researchers were unable to classify non-antibiotic medications in order to determine their significance in MRSA colonization. The major disadvantage of this study is that the cross-sectional data did not allow for a casualty investigation. It is uncertain if long-term antibiotics have a higher effect on *S aureus* colonization due to a lack of evidence on the duration and dose of antibiotics usage.^[34]^ Further studies are needed to determine the relationship between *S aureus* and the duration and dose of antibiotic use in the general population.

## 5. Conclusion

This is the first study to look at the connection between antibiotics and *S aureus* and MRSA colonization in a nationally representative cohort of patients. It demonstrates a low national prevalence of MRSA colonization but a widespread national prevalence of MSSA. Our findings indicated that broad-spectrum antibiotics were positively related to MSSA colonization. Antibiotics should be used in a controlled and suitable manner to reduce *S aureus* colonization. Physicians should be aware of the likelihood of MRSA colonization and the possibility of infection in patients who have used fluoroquinolone in the last 30 days. Former smokers should also practice better personal hygiene to limit the possibility of MRSA colonization.

## Author contributions

Baixing Chen study conception and design, acquisition of data, analysis and interpretation of data, writing manuscript; Shaoshuo Li, critical revision of manuscript; Shi Lin, critical revision of manuscript; Mingling Huang acquisition of data, critical revision of manuscript; Hang Dong study conception and design, drafting of manuscript, critical revision of manuscript.

**Conceptualization:** Baixing Chen, Hang Dong.

**Funding acquisition:** Hang Dong.

**Methodology:** Baixing Chen, Mingling Huang, Hang Dong.

**Software:** Baixing Chen.

**Writing – original draft:** Baixing Chen, Shaoshuo Li, Hang Dong.

**Writing – review & editing:** Shaoshuo Li, Shi Lin, Mingling Huang, Hang Dong.

## References

[1] von Eiff C Becker K Machka K. Nasal carriage as a source of *Staphylococcus aureus* bacteremia. Study Group. N Engl J Med. 2001;344:11–6. 11136954 10.1056/NEJM200101043440102

[2] O’Gara JP. Into the storm: chasing the opportunistic pathogen *Staphylococcus aureus* from skin colonisation to life-threatening infections. Environ Microbiol. 2017;19:3823–33. 28631399 10.1111/1462-2920.13833

[3] Klevens RM Morrison MA Nadle J. Invasive methicillin-resistant *Staphylococcus aureus* infections in the United States. JAMA. 2007;298:1763–71. 17940231 10.1001/jama.298.15.1763

[4] Hidron AI Edwards JR Patel J. NHSN annual update: antimicrobial-resistant pathogens associated with healthcare-associated infections: annual summary of data reported to the National Healthcare Safety Network at the Centers for Disease Control and Prevention, 2006-2007. Infect Control Hosp Epidemiol. 2008;29:996–1011. 18947320 10.1086/591861

[5] Karanika S Zervou FN Zacharioudakis IM. Risk factors for meticillin-resistant *Staphylococcus aureus* colonization in dialysis patients: a meta-analysis. J Hosp Infect. 2015;91:257–63. 26428959 10.1016/j.jhin.2015.07.014

[6] Popovich KJ Smith KY Khawcharoenporn T. Community-associated methicillin-resistant *Staphylococcus aureus* colonization in high-risk groups of HIV-infected patients. Clin Infect Dis. 2012;54:1296–303. 22354926 10.1093/cid/cis030 PMC3404690

[7] de Benito S Alou L Becerro-de-Bengoa-Vallejo R. Prevalence of Staphylococcus spp. nasal colonization among doctors of podiatric medicine and associated risk factors in Spain. Antimicrob Resist Infect Control. 2018;7:24. 29468052 10.1186/s13756-018-0318-0 PMC5816397

[8] Karanika S Kinamon T Grigoras C. Colonization with methicillin-resistant *Staphylococcus aureus* and risk for infection among asymptomatic athletes: a systematic review and metaanalysis. Clin Infect Dis. 2016;63:195–204. 27090988 10.1093/cid/ciw240

[9] Van Nguyen K Zhang T Thi Vu BN. *Staphylococcus aureus* nasopharyngeal carriage in rural and urban northern Vietnam. Trans R Soc Trop Med Hyg. 2014;108:783–90. 25187670 10.1093/trstmh/tru132 PMC4235569

[10] Sfeir M Obeid Y Eid C. Prevalence of *Staphylococcus aureus* methicillin-sensitive and methicillin-resistant nasal and pharyngeal colonization in outpatients in Lebanon. Am J Infect Control. 2014;42:160–3. 24360641 10.1016/j.ajic.2013.08.008

[11] Chen BJ Xie XY Ni LJ. Factors associated with *Staphylococcus aureus* nasal carriage and molecular characteristics among the general population at a Medical College Campus in Guangzhou, South China. Ann Clin Microbiol Antimicrob. 2017;16:28. 28399856 10.1186/s12941-017-0206-0 PMC5387264

[12] Moran GJ Krishnadasan A Gorwitz RJ. Methicillin-resistant *S. aureus* infections among patients in the emergency department. N Engl J Med. 2006;355:666–74. 16914702 10.1056/NEJMoa055356

[13] Gorwitz RJ Kruszon-Moran D McAllister SK. Changes in the prevalence of nasal colonization with *Staphylococcus aureus* in the United States, 2001-2004. J Infect Dis. 2008;197:1226–34. 18422434 10.1086/533494

[14] Halablab MA Hijazi SM Fawzi MA. *Staphylococcus aureus* nasal carriage rate and associated risk factors in individuals in the community. Epidemiol Infect. 2010;138:702–6. 19941687 10.1017/S0950268809991233

[15] Munckhof WJ Nimmo GR Schooneveldt JM. Nasal carriage of *Staphylococcus aureus*, including community-associated methicillin-resistant strains, in Queensland adults. Clin Microbiol Infect. 2009;15:149–55. 19154489 10.1111/j.1469-0691.2008.02652.x

[16] Miller M Cook HA Furuya EY. *Staphylococcus aureus* in the community: colonization versus infection. PLoS One. 2009;4:e6708. 19693269 10.1371/journal.pone.0006708 PMC2724739

[17] Davoodabadi F Mobasherizadeh S Mostafavizadeh K. Nasal colonization in children with community acquired methicillin-resistant *Staphylococcus aureus*. Adv Biomed Res. 2016;5:86. 27274501 10.4103/2277-9175.182217 PMC4879855

[18] Methicillin - Resistant *Staphylococcus aureus* (MRSA). Available at: https://wwwn.cdc.gov/Nchs/Nhanes/2003-2004/L35_C.htm [access date November 10, 2021].

[19] Lumley T. Complex surveys: a guide to analysis using R. Washington: John Wiley & Sons, 2011.

[20] Rahman HH Niemann D Munson-McGee SH. Environmental exposure to metals and the risk of high blood pressure: a cross-sectional study from NHANES 2015-2016. Environ Sci Pollut Res Int. 2022;29:531–42. 34331653 10.1007/s11356-021-15726-0

[21] Chen B Dai X He B. Differences in *Staphylococcus aureus* nasal carriage and molecular characteristics among community residents and healthcare workers at Sun Yat-Sen University, Guangzhou, Southern China. BMC Infect Dis. 2015;15:303. 26223250 10.1186/s12879-015-1032-7 PMC4520063

[22] Mehraj J Akmatov MK Strompl J. Methicillin-sensitive and methicillin-resistant *Staphylococcus aureus* nasal carriage in a random sample of non-hospitalized adult population in northern Germany. PLoS One. 2014;9:e107937. 25251407 10.1371/journal.pone.0107937 PMC4176714

[23] Lautenbach E Tolomeo P Nachamkin I. The impact of household transmission on duration of outpatient colonization with methicillin-resistant *Staphylococcus aureus*. Epidemiol Infect. 2010;138:683–5. 20109256 10.1017/S0950268810000099 PMC2847002

[24] Johansson PJ Gustafsson EB Ringberg H. High prevalence of MRSA in household contacts. Scand J Infect Dis. 2007;39:764–8. 17701713 10.1080/00365540701302501

[25] Huijsdens XW van Santen-Verheuvel MG Spalburg E. Multiple cases of familial transmission of community-acquired methicillin-resistant *Staphylococcus aureus*. J Clin Microbiol. 2006;44:2994–6. 16891525 10.1128/JCM.00846-06 PMC1594612

[26] Olsen K Falch BM Danielsen K. *Staphylococcus aureus* nasal carriage is associated with serum 25-hydroxyvitamin D levels, gender and smoking status. The Tromso Staph and Skin Study. Eur J Clin Microbiol Infect Dis. 2012;31:465–73. 21811869 10.1007/s10096-011-1331-x PMC3303067

[27] Sakr A Bregeon F Mege JL. *Staphylococcus aureus* nasal colonization: an update on mechanisms, epidemiology, risk factors, and subsequent infections. Front Microbiol. 2018;9:2419. 30349525 10.3389/fmicb.2018.02419 PMC6186810

[28] Wertheim HF Melles DC Vos MC. The role of nasal carriage in *Staphylococcus aureus* infections. Lancet Infect Dis. 2005;5:751–62. 16310147 10.1016/S1473-3099(05)70295-4

[29] Andreatos N Shehadeh F Pliakos EE. The impact of antibiotic prescription rates on the incidence of MRSA bloodstream infections: a county-level, US-wide analysis. Int J Antimicrob Agents. 2018;52:195–200. 29656062 10.1016/j.ijantimicag.2018.04.003

[30] Lacoma A Edwards AM Young BC. Cigarette smoke exposure redirects *Staphylococcus aureus* to a virulence profile associated with persistent infection. Sci Rep. 2019;9:10798. 31346202 10.1038/s41598-019-47258-6 PMC6658544

[31] Kulkarni R Caskey J Singh SK. Cigarette smoke extract-exposed methicillin-resistant *Staphylococcus aureus* regulates leukocyte function for pulmonary persistence. Am J Respir Cell Mol Biol. 2016;55:586–601. 27253086 10.1165/rcmb.2015-0397OC PMC5070110

[32] Couderc C Jolivet S Thiebaut AC. Fluoroquinolone use is a risk factor for methicillin-resistant *Staphylococcus aureus* acquisition in long-term care facilities: a nested case-case-control study. Clin Infect Dis. 2014;59:206–15. 24729496 10.1093/cid/ciu236

[33] Pogorzelska-Maziarz M Furuya EY Larson EL. Risk factors for methicillin-resistant *Staphylococcus aureus* bacteraemia differ depending on the control group chosen. Epidemiol Infect. 2013;141:2376–83. 23425708 10.1017/S0950268813000174 PMC4065413

[34] Tacconelli E De Angelis G Cataldo MA. Does antibiotic exposure increase the risk of methicillin-resistant *Staphylococcus aureus* (MRSA) isolation? A systematic review and meta-analysis. J Antimicrob Chemother. 2008;61:26–38. 17986491 10.1093/jac/dkm416

[35] Muto CA Jernigan JA Ostrowsky BE. SHEA guideline for preventing nosocomial transmission of multidrug-resistant strains of *Staphylococcus aureus* and enterococcus. Infect Control Hosp Epidemiol. 2003;24:362–86. 12785411 10.1086/502213

